# In-situ aerosol nanoparticle characterization by small angle X-ray scattering at ultra-low volume fraction

**DOI:** 10.1038/s41467-019-09066-4

**Published:** 2019-03-08

**Authors:** P. S. Bauer, H. Amenitsch, B. Baumgartner, G. Köberl, C. Rentenberger, P. M. Winkler

**Affiliations:** 1 University of Vienna, Faculty of Physics, 1090 Vienna, Austria; 2 Graz University of Technology, Institute of Inorganic Chemistry, 8010 Graz, Austria

## Abstract

State-of-the-art aerosol nanoparticle techniques all have one feature in common: for analysis they remove the nanoparticles from their original environment. Therefore, physical and chemical properties of the particles might be changed or cannot be measured correctly. To overcome these shortcomings, we apply synchrotron based small angle X-ray scattering (SAXS) as an in-situ measurement technique. Contrasting other aerosol studies using SAXS, we focus on particle concentrations which allow direct comparison to common aerosol nanoparticle analyzers. To this end, we analyze aerosol nanoparticles at ambient pressure and concentrations of slightly above ~10^6^ cm^−3^. A differential mobility particle sizer (DMPS) is operated in parallel. We find that SAXS enables measurement of the primary particles and the aggregates, whereas the DMPS detects only aggregates. We conclude that in-situ nanoparticle characterization with ultra-low volume fractions of ~10^–10^ is feasible with SAXS. Our technique opens up a doorway to the in-situ analysis of aerosol nanoparticles under atmospheric conditions.

## Abstract (web summary)

State‐of‐the‐art aerosol nanoparticle techniques are limited by the shortcomings of removing the nanoparticles from their original environment. Here, the authors apply small angle X‐ray scattering as an in‐situ measurement technique, enabling the measurement of the primary particles and the aggregates.

## Introduction

Experimental aerosol research dates back more than 100 years ago, when John Aitken built a device to count dust particles in the air^1^. He discovered that several meteorological phenomena (coloring of the sky, transparency, visibility) depend on the concentration of these particles and vice versa (e.g. amount of dust and the direction of wind)^2^. Since then the study of small airborne particles, so called aerosol particles, evolved quickly to an own interdisciplinary research subject that led to numerous discoveries in fundamental physics and revealed important insights to health and climate effects. Nowadays we are more aware of the consequences of particulate air pollution on our health^3^ or the impact of cloud microphysics on our weather and climate^4^. Still, one of the largest uncertainties in climate modeling depicts the aerosol contribution to the radiative forcing^5,6^. Especially, surface properties and structure of nanoparticles play an important role for the formation of clouds and ice^7^ and may thus significantly impact climate. For this reason, a lot of effort in current aerosol research is put into the evaluation of surface properties and structural information of nanoparticles.

Recent instrumental developments made it possible to measure physical and chemical properties of airborne particles of nearly arbitrary small sizes. Well-established techniques like condensation particle counters (CPC), similar to the Aitken dust particle counter, can nowadays detect aerosol particles as small as ~1 nm^8–10^. Similarly, particle size distribution measurements by differential or scanning mobility particle sizers (DMPS or SMPS) have been extended to molecular dimensions^11–13^.

Despite their great contributions to the understanding of aerosol dynamics, a common drawback of these techniques is that they remove the aerosol particles from their original environment. Thereby particle properties may be changed by the measurement system. Especially nanoparticles formed by gas-to-particle conversion might be strongly affected by the measurement systems since the gas surrounding the particles has a direct influence on their formation. This problem becomes evident in a differential mobility analyzer (DMA) where the parent gas phase of newly formed nanoparticles is replaced by the sheath air. In order to allow proper size analysis in a DMA, particles must also be electrically charged beforehand, which may add contaminants to the particles under investigation. In addition, the electrical mobility distribution contains neither information about the structure of the particles (spheres, rods, aggregates, etc.) nor on the surface properties. The situation is even more dramatic in mass spectrometers, where nanoparticles or their constituents are exposed to high vacuum. The remaining signal is far from giving a complete picture of nanoparticle composition. Furthermore, a large fraction of nanoparticles gets lost, e.g., by wall collisions inside the instruments, which may affect the measured size distribution and concentration^14^. Currently, such instrumental effects on nanoparticles are insufficiently characterized and not well understood. Thus, new ways of nanoparticle analysis are desirable, allowing in-situ characterization of size and shape without the need to condition particles for measurement purposes.

Small-angle X-ray scattering (SAXS) is capable of measuring in-situ structure and size of particles in the nanometer range^15–17^. SAXS is a well-established technique in material science or in biochemical process analysis^18–21^. The availability of high-intensity X-ray beams at synchrotrons made it possible to measure aerosol particles directly in the gas phase^22–24^.

While previous SAXS studies on aerosols were performed at low pressures or particle concentrations oftentimes exceeding 10^12^ cm^−3^, we aim for conditions with ambient pressure and particle concentrations well below 10^7^ cm^−3^. Such conditions allow in situ SAXS and DMPS measurements, respectively, in parallel. While these settings correspond to the upper concentration limit of conventional aerosol techniques, the low volume fraction of the nanoparticles coincides with the lower detection limit of synchrotron-based SAXS. The purpose of this study is to close the gap in the measurement range of these techniques. Therefore, we designed a measurement system consisting of a flow reactor that can be placed directly in the SAXS beam and measure in parallel with conventional aerosol analyzers.

## Results

### Experimental procedure

**Fig. 1:**
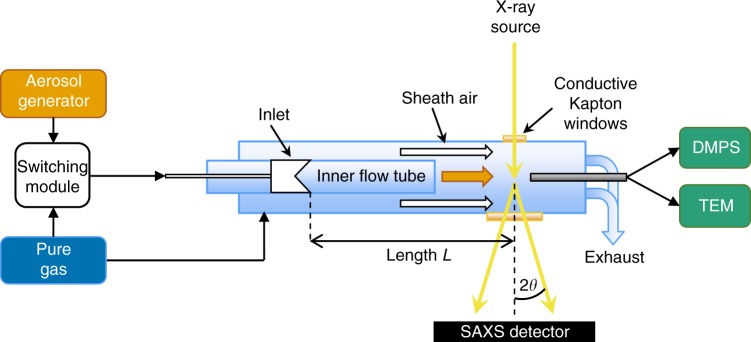
Schematic drawing of the flow tube reactor at the SAXS beamline. Either particle-laden air from the aerosol generator or pure air is injected through the moveable inlet into the inner flow tube. The sheath air system and the conductive Kapton are precautions against particle deposition on the windows. The particles are measured on the one hand with the SAXS system and on the other hand with conventional aerosol measurement techniques (DMPS and TEM) after core sampling

Aerosol flow reactors are a widely used tool in aerosol science to study dynamical effects of aerosols within the range of ambient conditions^25–27^. Figure 1 illustrates a schematic of the flow tube setup used in this study. Further details on the setup are described in the Methods section. The SAXS experiments were conducted at the Austrian SAXS beamline at ELETTRA, Trieste, Italy^28^ and at the ID02 beamline at the European Synchrotron Radiation Facility (ESRF), Grenoble, France^29^.

An important parameter for SAXS is the scattering angle *θ*, which is related to the length of the scattering vector via $q = \left| {\vec q} \right| = (4\pi /\lambda ){\mathrm{sin}}(\theta /2)$, where *λ* is the wavelength^30,31^. The data reduction was done by the freely available software of the beamlines. The two-dimensional data were circularly averaged to convert them into one-dimensional scattering curves. Further data analysis, i.e. background subtraction and curve fitting, was carried out with the software package Igor Pro and the use of the macro package IRENA^32^.

### Reduction of SAXS background

A critical step in our SAXS analysis is the extraction of the actual scattering signal from background effects evolving from the system or the surrounding gas. Since the scattering contrast of our sample is close to the limit of detection we designed a differential background subtraction (DBS) method where SAXS signals were acquired alternatingly from the aerosol and particle-free carrier gas, respectively. In our setup, several issues of background effects were addressed, some of which are discussed in the Supplementary Methods. The most important advances, the DBS method and the use of helium as carrier gas, are discussed below.

**Fig. 2:**
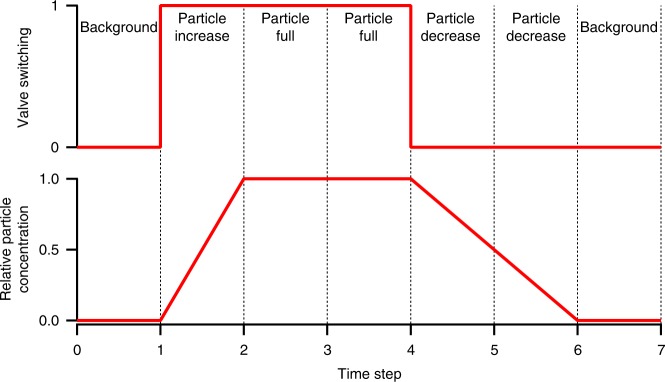
The seven-step procedure of the differential background subtraction method. The upper graph shows the switching cycle between pure gas (zero in the diagram) and particle-laden gas (one in the diagram) of the switching module. The lower diagram displays the theoretically expected relative particle concentration in the flow tube. Since it takes longer to diminish the particle concentration in the flow tube by flushing with pure gas, the decrease phase was scheduled with two time steps. The different phases are marked in the upper section. The time steps with full particle concentration are reserved for the actual SAXS measurements

The idea behind the DBS method is to acquire a background before and after each measurement with particle-laden air, average them, and take it as background for the data. This procedure provides a certain stability against deposition and beam fluctuations (see SI for more information). Figure 2 illustrates the switching cycle between particle-free gas (pure gas) and particle-laden gas (aerosol) for background and particle measurements, respectively. The switching module was synchronized with the SAXS acquisition system. We introduced a seven-step cycle to provide enough time for the particle concentration to build up and to flush the flow tube. After the first background with pure gas only, the switching module injects particles into the flow tube. Following the particle concentration build-up phase, two time steps with full particle concentration are allocated for SAXS particle measurements. Then the switching module again flushes the flow tube with pure gas to get rid of the particles for two time steps. The last step of the routine is another background, which will be averaged with the first background. This cycle is repeated several times to provide reasonable statistical validation.

**Fig. 3:**
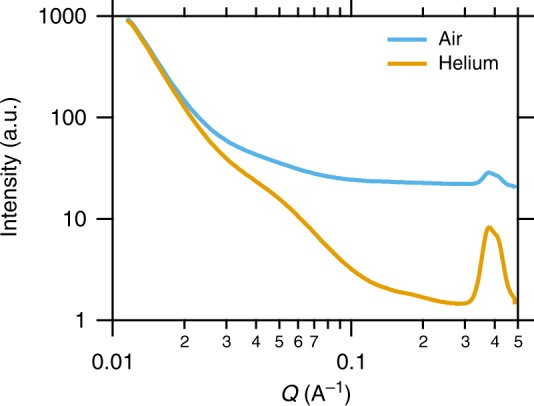
Comparison of the raw SAXS signal of pure air and helium measured at ELETTRA. The background for helium is lower due to its lower electron density. In the low *q*-limit the scattering of the conductive Kapton is prevailing. In the intermediate and high *q*-range the signal from helium is about one order of magnitude lower than that of air. Thus, in the high *q*-range the Kapton peak is much more pronounced with helium

In order to minimize scattering effects of the carrier gas common air was replaced by helium. Helium is a noble gas and has only two electrons per atom, which gives a very low electron density compared to air thus reducing the background considerably. The real part scattering length density (SLD) (a measure for the electron density of the material) ratio of air to helium is about 15:2 under normal temperature and pressure conditions. Figure 3 illustrates a comparison between air and helium background taken at the SAXS beamline at ELETTRA. Especially in the high *q*-range the difference is about one order of magnitude in scattering intensity. Therefore, the Kapton peak (between *q* = 3 and 5 nm^−1^) is clearly visible in helium. In the low *q*-range the scattering of the Kapton windows is the limiting factor. Importantly, the helium flow is under ambient pressure and conventional aerosol instruments were used for reference measurements. Aerosol studies with a DMPS in ambient pressurized helium were already successfully conducted^33^. The DMPS system was thoroughly tested and characterized with helium in the lab.

### SAXS results

**Fig. 4:**
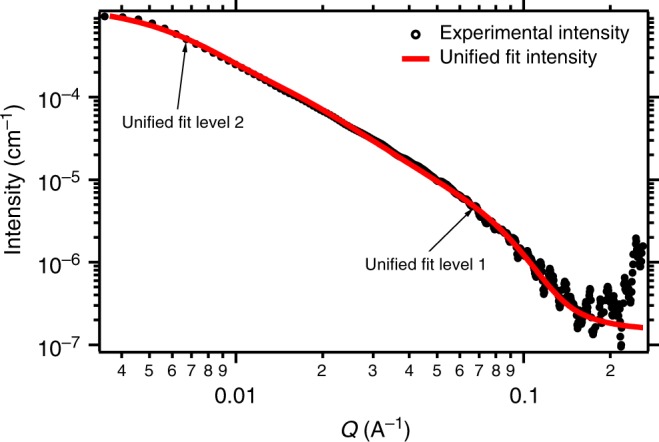
SAXS curve of tungsten blue oxide particles in helium measured at ESRF. The signal is derived by averaging the SAXS curves after applying the differential background subtraction (DBS). A unified two-level Beaucage fit (red line) nicely reproduces the shape of the SAXS curve. The coefficients of the fit are reported in Table 1. Additionally the position of structural levels 1 and 2 are indicated in the graph as well

Based on these two important advances, it was possible to gather SAXS signals at ESRF from tungsten blue oxide^34^ nanoparticles in helium, which are displayed in Fig. 4. The intensity of the scattering curve was calibrated with water^35^ and scaled to the diameter of the flow tube resulting in an absolute intensity scale in cm^−1^. The black dots represent the data resulting from the DBS averaging and the red curve a unified fit according to Beaucage^36,37^ which is discussed in detail in the Methods section. The fit here is a two-level Beaucage fit, where the first level represents the primary particles and the second level the aggregates. As described in the paper about mass-fractral aggregate SAXS measurements^38^, it is possible to measure directly the fractal dimension of aggregates with SAXS, which corresponds to the power-law regime slope $(I(q)\sim q^{ - d_{\mathrm{f}}})$ in the range between $\frac{1}{{R_2}} \, < \, q \, < \, \frac{1}{{R_1}}$. The limiting sizes of the primary particles (*R*_1_) and the aggregates (*R*_2_) can be linked to the mass ratio $z = \frac{{M_2}}{{M_1}}$ with the fractal dimension *d*_f_ as exponent (described in detail in the Methods section). The fit parameters of both levels are displayed in Table 1, the resulting parameters for the primary particles in Table 2, and the results for the aggregates in Table 3. To get a stable fit, the fractal dimension of the aggregates was fitted beforehand and fixed to *d*_f,2_ = 1.9.

**Table 1:** Coefficients of the two-level unified fit of Fig. 4

Level	*G* [cm^−1^]	*R*_g_ [nm]	*B*	*P* or *d*_f_	
1	1.57e−05 ± 4.43e−08	2.92 ± 0.003	5.25e−11 ± 2.48e−13	4.0 (fixed)	Background 1.5e−07 (fixed)
2	1.31e−03 ± 1.34e−05	28.11 ± 0.23	4.65e−08 ± 1.07e−10	1.9 (fixed)	RgCO = 50[A]

**Table 2:** Results of the size distribution analysis using unified fit results of level 1

PDI	*m* [nm]	〈*R*〉 [nm]	*σ*
1.509	2.97	3.02	0.185

**Table 3:** Results of the branched mass-fractal analysis using unified fit results of both levels

*z*	*d*_min_	*c*	*ϕ*_Br_
83.8	1.543	1.231	0.565

From the first level of the unified fit it is possible to directly calculate the polydispersity index (PDI) resulting in the momenta of a log-normal distribution for the primary particles^39^ as described in detail in the Methods section. The first level radius of gyration *R*_g,1_ = 2.92 nm and the other parameters result in a PDI = 1.51, a mean radius of *r*_prim_ = 3.02 nm and a standard deviation of *σ* = 0.19. The primary particles have therefore a diameter around *d*_prim_ = 6.04 nm. Together with the second level, the mass ratio of aggregates to primary particles $z = \frac{{M_2}}{{M_1}}$ is calculated, which can be seen as number of primary particles per aggregate. In our case there are in average about *z* = 83.8 primary particles per aggregate. The scaling law for mass-fractal aggregates gives then the average size of the aggregate with *R*_2_ = 31.1 nm and an average aggregate diameter of *D*_2_ = 62.2 nm. These results can be compared to images from a transmission electron microscope (TEM).

### TEM results

**Fig. 5:**
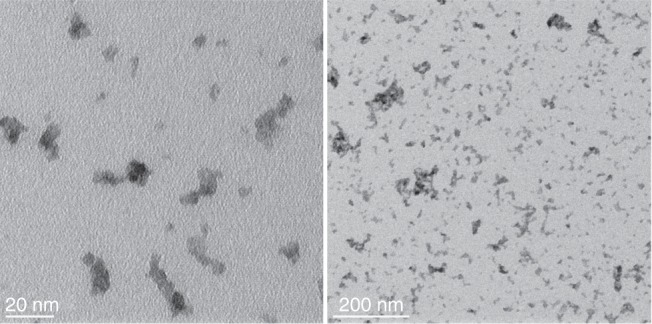
TEM images with different magnification of tungsten blue oxide aggregates. TEM samples were taken in parallel to the SAXS measurements. The primary particles have a mean diameter of about 5–6 nm. From the second image (lower magnification) it is plausible that the aggregates consist in average of about 84 primary particles and have an average size of about 62 nm

TEM samples were taken in parallel to the SAXS measurements. Two representative TEM images with different magnifications are shown in Fig. 5 to emphasize primary particles and aggregates, respectively. The TEM images were acquired with a Philips CM200 TEM equipped with a Gatan Orius CCD camera. In the first image (higher magnification) the primary particles are clearly visible showing an almost spherical shape (smooth surface) with a mean diameter of about 5–6 nm. The second image (lower magnification) is more an overview over a larger area to illustrate the aggregates. Thereby it becomes clear that a two-dimensional projection of an aggregate cannot gather all the information of the three-dimensional structure. In addition, it is extremely challenging to get relevant statistical data from a TEM image since only a small region of the sample is depicted. However, the interpretation of the overview image makes the SAXS results plausible, which yielded an average number of primary particles per aggregate of *z* = 83.8 and an average aggregate size of *D*_2_ = 62.2 nm. The information resulting from the TEM images on the individual particles hence support the statistical results gathered with SAXS.

### Volume fraction analysis

Another important SAXS parameter, which can be utilized due to the absolute intensity calibration, is the Porod invariant *Q*^31^. The Porod invariant is defined by

1 $$Q \equiv \mathop {\int }\nolimits_0^\infty q^2I\left( q \right){\mathrm d}q = 2\pi ^2\left( {{\mathrm{\Delta }}\rho } \right)^2\phi _{\mathrm{V}}\,\left( {1 - \phi _{\mathrm{V}}} \right) \approx 2\pi ^2\left( {{\mathrm{\Delta }}\rho } \right)^2\phi _{\mathrm{V}},$$

where *q* is the scattering vector, (Δ*ρ*)^2^ the electron density difference squared, and *ϕ*_V_ the volume fraction of the sample. For very low volume fractions, as in our case, the term (1 − *ϕ*_V_) is very close to one and can be neglected. The Porod invariant is very useful since it is derived from fundamental principles and can be used whenever the intensity is absolutely calibrated. The electron density was calculated for tungsten blue oxide (mass density of 7.16 g cm^−3^ (ref. ^40^)) in difference to helium resulting in a squared electron density of (Δ*ρ*)^2^ = 2.36 × 10^23^ cm^−4^. From the calculated Porod invariant of *Q* = 2.89 × 10^15^ cm^−4^ we obtain a volume fraction of *ϕ*_V_ = 6.2 × 10^−10^. This agrees with the estimates calculated beforehand and is comparable to the volume fraction calculated from the size distribution of the DMPS below.

### DMPS results

**Fig. 6:**
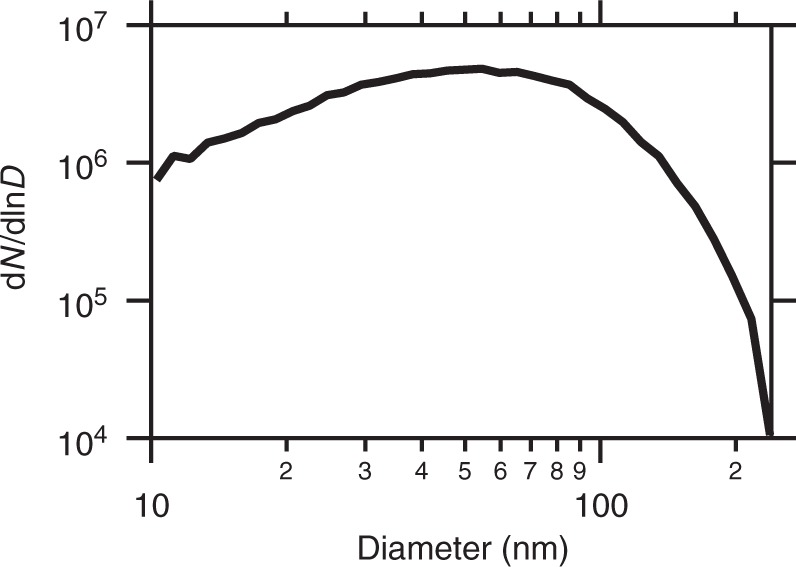
DMPS size distributions of tungsten blue oxide particles measured in helium. The shape of the curve does not follow a log-normal distribution, which indicates that the particles are aggregated. Several parameters can be derived from the distribution. The mode of the distribution is at *D*_mode_ = 54.5 nm and the mean (mobility) diameter is 〈*D*〉 = 52.5 nm. The geometric standard deviation result in *σ*_geom_ = 1.88 and the total concentration in *N*_conc_ = 3.47 × 10^6^ cm^−3^

Figure 6 shows the number size distribution measured with the DMPS system in helium. The distribution deviates from a log-normal distribution and is especially elevated for smaller particles. This indicates that the particles consist of aggregates and are formed by smaller particles. Several parameters of this number size distribution are determined. The mode of the distribution is at *D*_mode_ = 54.5 nm and the mean (mobility) diameter is determined to be 〈*D*〉 = 52.5 nm. The geometric standard deviation results in *σ*_geom_ = 1.88. In addition, the total concentration was calculated from the size distribution by integrating over *d*(ln *D*) and gives a concentration of 3.47 × 10^6^ cm^−3^. The volume fraction calculated from the distribution is *ϕ*_V_ = 6.46 × 10^−10^, which agrees very well with the volume fraction calculated with SAXS. The difference between the mean particle sizes of the DMPS and SAXS arises from the measurement approach. On the one hand, the diameter from a DMPS measurement is in principle the equivalent diameter of a sphere, which has the same electrical mobility as the particle. Mass-fractal aggregates, for example, can align with the air flow and might therefore appear smaller than determined from the scaling laws with SAXS. On the other hand, SAXS emphasizes the larger particles as explained in more detail in the Methods section.

## Discussion

To conclude, our study proves that SAXS is suitable to characterize aerosolized nanoparticles even at volume fractions as low as ~10^−10^. We have succeeded in determining nanoparticle size and structure from complementary instruments and closed the experimental gap between conventional aerosol nanoparticle analyzers and SAXS. Here we presented in situ SAXS measurements of aerosol nanoparticles in the concentration range of about ~10^6^ cm^−3^. The experiments were conducted in a closed flow tube, which was operated under ambient pressure and room temperature. We managed to reduce particle deposition at the windows significantly by using a sheath air system and conductive Kapton as window material. The low scattering signal of the particles compared to the background was enhanced by using helium as carrier gas. For stability reasons the system was operated in a DBS mode. During the measurement series taken over the course of a day, the switching between particle-free background and particle-laden helium facilitates adjustments to small changes in the beam intensity. It was possible to deduce SAXS scattering curves by averaging for every step of the procedure. The SAXS analysis was done with a two-level unified Beaucage fit, which yielded parameters of the mass-fractal aggregates and from the primary particles. The mean primary particle diameter of about 6 nm and the fractal dimension *d*_f_ = 1.9 of the aggregates result in a mean size of the aggregates of about 62 nm. In comparison, the DMPS measurements can only resolve the aggregates yielding a mean particle (mobility) diameter of about 52 nm. TEM images of the tungsten blue oxide aggregates support the results and complete the picture. This demonstrates that a combination of techniques (SAXS, DMPS, TEM) is decisive to get a more complete understanding of nanoparticle properties.

In terms of the analysis of low-intensity SAXS signals the DBS method can be considered another significant step forward. It should be noted though that the reduction of background scattering by the use of helium and the DBS method were obligatory to achieve these results. For future investigations it is of importance to use window materials, which have a lower scattering background especially in the low *q*-range. In addition, high and extremely stable beam intensities as available from latest generation synchrotrons are crucial to resolve the ultra-low volume fraction nanoparticles. Eventually, with these advances our technique will allow in situ analysis of aerosol nanoparticles in air under real atmospheric conditions.

## Methods

### Aerosol flow reactor setup

The setup, shown in Fig. 1, consists of two concentrically located tubes. The inner tube with a diameter of 22 mm constitutes the actual reactor and is equipped with an inlet, which is placed at a distance *L* (about 7 cm) to the measurement point, where the SAXS beam enters the flow tube. The inlet of the flow tube is connected to a switching module, feeding alternatingly particle-laden gas and particle-free gas into the flow tube, respectively. As aerosol source a hot wire generator^41,42^ with a tungsten wire was used. The outer flow tube with a diameter of 40 mm is connected to particle-free gas which is laminar superimposed with the inner flow about 1 cm before the measurement point. This is necessary to protect the Kapton windows from aerosol deposition. In addition, the 50-µm-thick aluminum-coated Kapton foil is electrically conductive to prevent particle deposition by electrostatic charging of the foil.

The SAXS beam enters the flow tube perpendicularly through the first Kapton window, with a diameter of 5 mm, gets scattered at the aerosol, and exits the flow tube via the second Kapton window, with a diameter of 16 mm. About 2 cm downstream of the SAXS measurement point particles are sampled with a centered stainless-steel tube and measured with a DMPS system. In addition, TEM samples are taken with a nanometer aerosol sampler (TSI 3089). These conventional aerosol techniques are for comparison purpose to the SAXS measurements. The remaining flow behind the core sampling is guided into the exhaust to keep the flow laminar inside the flow tube.

The flow tube is operated under standard laboratory conditions (ambient temperature around 25 °C and pressure of about 1 atm) at flow rates of about 3 lpm. Flow simulations of sheath air and core sampling were carried out and indicate laminar flow under regular operating conditions and therefore stable measurement conditions in the tube.

### SAXS analysis of mass-fractal aggregates

For dilute systems as in our case, SAXS is a challenging task to acquire good signal strength from the particles, especially in the high *q*-range. The scattering intensity in the high *q*-range is linked to the smaller particles, which have a lower signal. The SAXS signal is proportional to the volume squared and hence proportional to *R*^6^ for spherical particles. This imbalance in the weighting of the particle sizes makes it difficult to use direct fit function with certain predefined size distributions, since larger particles are always emphasized. This is especially true if the size distribution is rather broad and covers a large size range or consists of aggregates. A global scattering function utilizes general behaviors of SAXS curves and therefore includes the decline in the higher *q*-range. From the parameters of the global scattering function parameters of aggregates and a PDI can be derived, which can be linked to a particle size distribution for the primary particles.

In general, the SAXS spectrum can be divided into two regimes for each level, the power-law regime and the Guinier region. In the power-law regime in the higher *q*-range (*q* ≫ 1/*R*, with *R* as characteristic dimension of the particles) the particle surface scattering is the prevailing mechanism and the intensity can be described by a power law^38^

2 $$\begin{array}{*{20}{c}} {I\left( q \right) = Bq^{ - {{P}}},} \end{array}$$

where *B* is a constant prefactor and *P* the exponent. The power of *P* = 4 is only valid for smooth and sharp interfaces in the high *q*-range (Porod’s regime), which is the case for the scattering function of the primary particles. The factor *B* contains the surface to volume ratio *S*/*V* of the particles. At the limit of the surface scattering, in the low *q*-range (*q* ≪ 1/*R*), the Guinier region is described by^30^

3 $$\begin{array}{*{20}{c}} {I\left( q \right) = G\,{\mathrm{exp}}\left( { - \frac{{q^2R_{\mathrm{g}}^2}}{3}} \right),} \end{array}$$

where the factor *G* is proportional to the number density of the particles and the average squared volume. The radius of gyration *R*_g_ is a measurement for the average particle size and can be seen as the mass weighted root-mean-squared radius of the particle size distribution^43^.

These two regions are combined in the global unified scattering function, often referred to as Beaucage function^36,37^,

4 $$\begin{array}{*{20}{c}} {I\left( q \right) = G\,{\mathrm {exp}}\left( { - \frac{{q^2R_{\mathrm{g}}^2}}{3}} \right) + B\left( {q^ \ast } \right)^{ - P},\;q^ \ast = \frac{q}{{\left[ {{\mathrm{erf}}\left( {qR_{\mathrm{g}}/\sqrt 6 } \right)} \right]^{ - 3}}},} \end{array}$$

where erf( ) is the error function and *G*, *R*_g_ and *B*, *P* are the factors from the Guinier and power-law region. Equation (3) describes only one single level of structure, like polydisperse spherical particles if *P* = 4. The structural levels can be increased by a combination of these functions to fit mass-fractal aggregates, which is described in refs. ^37,44,45^. In our case, the SAXS curve indicates that the particles are aggregated because a second level is needed to fit the shape of the scattering curve. The power-law regime of the second level *P*_2_ is equal to the mass-fractal dimension *d*_f,2_ of the aggregates *P*_2_ = *d*_f,2_. The fit function for mass-fractal aggregates was published by Beaucage and Schaefer^46^. From the seven parameters of the fit function (*G*_1_, *R*_g1_, *B*_1_ and *G*_2_, *R*_g2_, *B*_2_, *d*_f,2_) the size distribution of the primary particles (indicated by subscript “1”), with *P*_1_ = 4, and parameters of aggregates (indicated by subscript “2”) are calculated.

For the primary particles (parameters *G*_1_, *R*_g1_ and *B*_1_ with *P*_1_ = 4) a PDI is derived by

5 $$\begin{array}{*{20}{c}} {{\mathrm {PDI}} = \frac{{B_1\,R_{{\mathrm{g}}1}^4}}{{1.62\,G_1}},} \end{array}$$

which is a convenient parameter to characterize the size distribution of the primary particles. Beaucage et al.^39^ showed that the PDI and the radius of gyration *R*_g1_ can be linked to the momenta of a log-normal distribution by

6 $$\begin{array}{*{20}{c}} {m = \sqrt {\frac{5}{3}} R_{{\mathrm{g}}1}{\mathrm{exp}}\left( { - 14\frac{{\sigma ^2}}{2}} \right),\quad \sigma = \sqrt {\frac{{\ln \left( {{\mathrm {PDI}}} \right)}}{{12}}} ,} \end{array}$$

where *m* is the median radius, which can be linked to the mean radius 〈*R*〉 together with the standard deviation *σ*. For monodisperse spheres the PDI would give PDI = 1 and therefore *σ* = 0 and the radius of the spheres will be given by $R = \sqrt {5/3} \,R_{{\mathrm{g}}1}$, which fits perfectly with the theory^30,31,47^.

The mass-fractal parameters are derived from a combination of the first- and second-level parameters^38^. A mass-fractal has two distinct sizes, defined by the primary particles (*R*_1_) and the aggregates (*R*_2_). The relative size of the aggregate to the primary particles $r = \frac{{R_2}}{{R_1}}$ can be linked to the fraction of the mass $z = \frac{{M_2}}{{M_1}}$ by a scaling relation

7 $$\begin{array}{*{20}{c}} {z = \alpha \cdot r^{d_{\mathrm{f}}},} \end{array}$$

where *α* is a constant (the lacunarity) and mostly close to 1 (depending on the growth mechanism). The mass-fractal dimension *d*_f_, explained in Eq. (2) with *P*_2_ = *d*_f,2_, is directly fitted in the second-level Porod region. The mass ratio *z* can also be seen as average number of primary particles inside an aggregate and is calculated by the ratio of the Guinier factors $z = \frac{{G_2}}{{G_1}}$. Other parameters resulting of the combination of the two levels of the unified fit are the connectivity dimension *c* (also known as intrinsic dimension^48^), the minimum dimension *d*_min_, and the number fraction of branches *ϕ*_Br_. These parameters are described in detail in the paper from Beaucage^38^, but will not be discussed further here.

## Supplementary information

Supplementary Information

Peer Review File

## Data Availability

All data needed to evaluate the conclusions in the paper are present in the paper and/or the Supplementary Information. Additional data related to this paper may be requested from the corresponding author.

## References

[1] Aitken, J. On a simple pocket dust-counter. *Proc. R. Soc. Edinb. (1890–1891)* **18**, 39–52 (1891).

[2] Aitken J (1894). Dust and meteorological phenomena. Nature.

[3] WHO. *Ambient Air Pollution: A Global Assessment of Exposure and Burden of Disease* (WHO, 2016).

[4] Stubenrauch CJ (2013). Assessment of global cloud datasets from satellites: project and database initiated by the GEWEX radiation panel. Bull. Am. Meteorol. Soc..

[5] IPCC. *Climate Change 2013: The Physical Science Basis.**Contribution of Working Group I to the Fifth Assessment Report of the Intergovernmental Panel on Climate Change* (eds. Stocker, T. F. et al.) (Cambridge University Press, Cambridge, 2013).

[6] Carslaw KS (2013). Large contribution of natural aerosols to uncertainty in indirect forcing. Nature.

[7] Laaksonen A, Malila J, Nenes A, Hung HM, Chen JP (2016). Surface fractal dimension, water adsorption efficiency and cloud nucleation activity of insoluble aerosol. Sci. Rep..

[8] Winkler PM (2008). Heterogeneous nucleation experiments bridging the scale from molecular ion clusters to nanoparticles. Science.

[9] Iida K, Stolzenburg MR, McMurry PH (2009). Effect of working fluid on sub-2 nm particle detection with a laminar flow ultrafine condensation particle counter. Aerosol Sci. Technol..

[10] Vanhanen J (2011). Particle size magnifier for nano-CN detection. Aerosol Sci. Technol..

[11] Brunelli NA, Flagan RC, Giapis KP (2009). Radial differential mobility analyzer for one nanometer particle classification. Aerosol Sci. Technol..

[12] Steiner G, Attoui M, Wimmer D, Reischl GP (2010). A medium flow, high-resolution Vienna DMA running in recirculating mode. Aerosol Sci. Technol..

[13] Mora JF, Kozlowski J (2013). Hand-held differential mobility analyzers of high resolution for 1–30 nm particles: design and fabrication considerations. J. Aerosol Sci..

[14] Wang J, Flagan RC, Seinfeld JH (2002). Diffusional losses in particle sampling systems containing bends and elbows. J. Aerosol Sci..

[15] Beaucage G (2004). Probing the dynamics of nanoparticle growth in a flame using synchrotron radiation. Nat. Mater..

[16] Mitchell JBA (2008). Evidence for nanoparticles in microwave-generated fireballs observed by synchrotron X-ray scattering. Phys. Rev. Lett..

[17] Wyslouzil BE, Wilemski G, Strey R, Seifert S, Winans RE (2007). Small angle X-ray scattering measurements probe water nanodroplet evolution under highly non-equilibrium conditions. Phys. Chem. Chem. Phys..

[18] Lipfert J, Doniach S (2007). Small-angle X-ray scattering from RNA, proteins, and protein complexes. Annu. Rev. Biophys. Biomol. Struct..

[19] Narayanan T (2009). High brilliance small-angle X-ray scattering applied to soft matter. Curr. Opin. Colloid Interface Sci..

[20] Mertens HDT, Svergun DI (2010). Structural characterization of proteins and complexes using small-angle X-ray solution scattering. J. Struct. Biol..

[21] Svergun DI, Koch MHJ (2003). Small-angle scattering studies of biological macromolecules in solution. Rep. Prog. Phys..

[22] Mitchell JBA (2002). X-ray synchrotron radiation probing of an ethylene diffusion flame. Combust. Flame.

[23] Sen D, Spalla O, Taché O, Haltebourg P, Thill A (2007). Slow drying of a spray of nanoparticles dispersion. In situ SAXS investigation. Langmuir.

[24] Laksmono H (2011). Monomer, clusters, liquid: an integrated spectroscopic study of methanol condensation. Phys. Chem. Chem. Phys..

[25] Shrestha M, Zhang Y, Ebben CJ, Martin ST, Geiger FM (2013). Vibrational sum frequency generation spectroscopy of secondary organic material produced by condensational growth from α-Pinene ozonolysis. J. Phys. Chem. A.

[26] Jungnikl K (2011). Aerosol flow reactor with controlled temperature gradient for in situ gas-phase X-ray experiments—measurements of Evaporation-Induced Self-Assembly (EISA) in aerosols. Aerosol Sci. Technol..

[27] Winkler PM (2012). Identification of the biogenic compounds responsible for size-dependent nanoparticle growth. Geophys. Res. Lett..

[28] Amenitsch H (1998). First performance assessment of the small-angle X-ray scattering beamline at ELETTRA. J. Synchrotron Radiat..

[29] Van Vaerenbergh P (2016). An upgrade beamline for combined wide, small and ultra small-angle x-ray scattering at the ESRF. AIP Conf. Proc..

[30] Guinier, A. & Fournet, G. *Small-Angle Scattering of X-Rays* (John Wiley & Sons, New York, 1955).

[31] Glatter, O. & Kratky, O. *Small Angle X-Ray Scattering* (Academic Press, London, 1982).

[32] Ilavsky J, Jemian PR (2009). Irena: tool suite for modeling and analysis of small-angle scattering. J. Appl. Crystallogr..

[33] Maisser A, Barmpounis K, Attoui MB, Biskos G, Schmidt-Ott A (2015). Atomic cluster generation with an atmospheric pressure spark discharge generator. Aerosol Sci. Technol..

[34] Lassner, E. & Schubert, W.-D. in *The Chemistry of Non-Sag Tungsten* (eds Bartha, L. et al.) 111–117 (Pergamon, Oxford, 1995).

[35] Orthaber D, Bergmann A, Glatter O (2000). SAXS experiments on absolute scale with Kratky systems using water as a secondary standard. J. Appl. Crystallogr..

[36] Beaucage G (1995). Approximations leading to a unified exponential/power-law approach to small-angle scattering. J. Appl. Crystallogr..

[37] Beaucage G (1996). Small-angle scattering from polymeric mass fractals of arbitrary mass-fractal dimension. J. Appl. Crystallogr..

[38] Beaucage G (2004). Determination of branch fraction and minimum dimension of mass-fractal aggregates. Phys. Rev. E.

[39] Beaucage G, Kammler HK, Pratsinis SE (2004). Particle size distributions from small-angle scattering using global scattering functions. J. Appl. Crystallogr..

[40] American Elements. *American Elements Tungsten Blue Oxide*. https://www.americanelements.com/blue-tungsten-oxide-tbo (2018).

[41] Boies AM, Lei P, Calder S, Shin WG, Girshick SL (2011). Hot-wire synthesis of gold nanoparticles. Aerosol Sci. Technol..

[42] Peineke C, Attoui MB, Schmidt-Ott A (2006). Using a glowing wire generator for production of charged, uniformly sized nanoparticles at high concentrations. J. Aerosol Sci..

[43] Li T, Senesi AJ, Lee B (2016). Small angle X-ray scattering for nanoparticle research. Chem. Rev..

[44] Mulderig A, Beaucage G, Vogtt K, Jiang H, Kuppa V (2017). Quantification of branching in fumed silica. J. Aerosol Sci..

[45] di Stasio S (2017). Soot with 1013 cm^−3^ high concentration and 25 Å radius of gyration as detected by small-angle X-ray scattering in a premixed ethylene-air flame at sooting threshold. J. Aerosol Sci..

[46] Beaucage G, Schaefer DW (1994). Structural studies of complex systems using small-angle scattering: a unified Guinier/power-law approach. J. Non-Cryst. Solids.

[47] Feigin, L. A. & Svergun, D. L. *Structure Analysis by Small-Angle X-Ray and Neutron Scattering* (D.I. Plenum Press, New York, 1987).

[48] Meakin P, Majid I, Havlin S, Stanley HE (1984). Topological properties of diffusion limited aggregation and cluster-cluster aggregation. J. Phys. A Math. Gen..

