# Supplementary material for: In-situ aerosol nanoparticle characterization by small angle X-ray scattering at ultra-low volume fraction
Source: Nat Commun. 2019 Mar 8;10:1122. doi: 10.1038/s41467-019-09066-4 (PMC6408461; doi:10.1038/s41467-019-09066-4)
Supplement: Supplementary file 1 — Supplementary Information [file 41467_2019_9066_MOESM1_ESM.pdf]

Supplementary Information: In-situ aerosol nanoparticle characterization by small angle x-ray scattering at ultra-low volume fraction

P.S. Bauer et al.

Supplementary Methods

In this supplementary information different SAXS background effects, like particle deposition, beam instabilities and carrier gas are discussed. These effects have been taken into account to build a setup suitable for in-situ nanoparticle characterization with SAXS. Here we give some insight to the pitfalls and progress made during our SAXS studies.

In order to obtain SAXS signals at decent signal-to-noise ratios from samples with ultra-low volume fraction it is mandatory to account for background effects, like deposition at the windows or beam instabilities. Our first attempt with SAXS measurements taken at the ELETTRA synchrotron shows substantial particle deposition at the Kapton windows as no precautions with respect to deposition were undertaken at the beginning. The integrated intensity of the measurement series is displayed in Supplementary Figure 1, where the effect of rising intensity due to deposition can be seen. During the measurements the Kapton windows and the particles get electrostatically charged by the x-ray beam and thus the charged windows attract the nanoparticles basically instantly due to their high electrical mobility. In the end, the second background with no particles in the flow tube has the highest intensity because of the deposited particles at the windows.

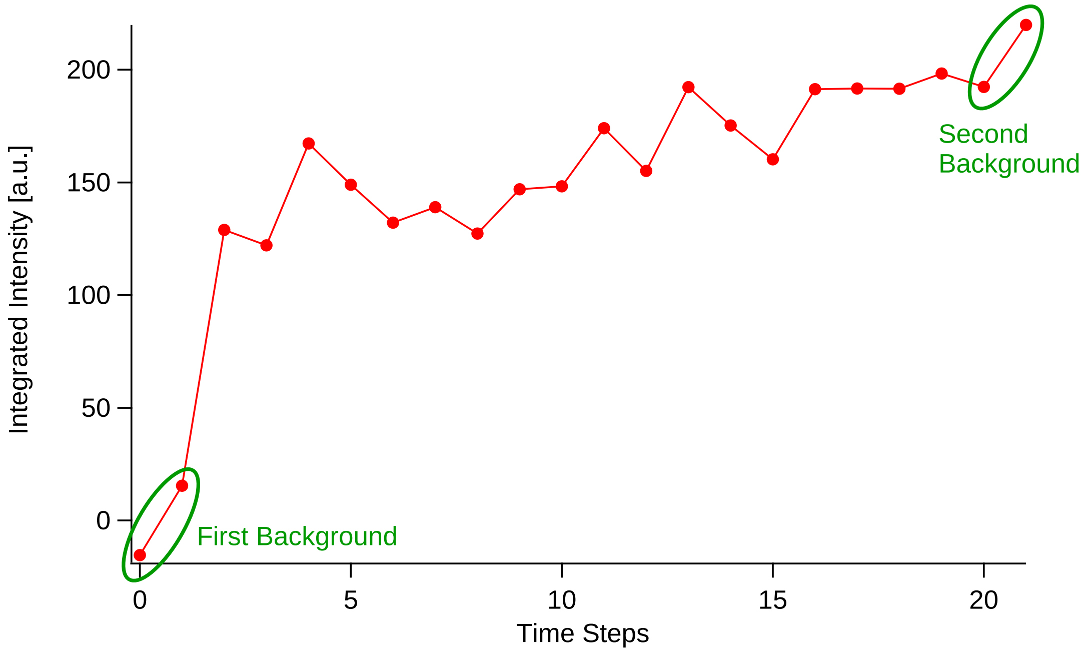

Supplementary Figure 1. **Integrated SAXS intensity of our first measurement series with substantial deposition at the windows.** The integrated intensity represents the total particle concentration in the beam. The first background with particle free air scatters around zero. Then, particles were inserted into the flow tube and the intensity is rising. At the end, another background with particle-free air was taken as reference. The constant rise of the integrated intensity (even for the second background) denotes for substantial deposition at the windows of the flow tube.

Several actions have been taken to deal with the background effects. To avoid deposition at the windows a sheath air system around the actual particle stream was applied and aluminum coated, conductive Kapton windows were installed. A flow simulation, shown in Supplementary Figure 2, confirms stable and laminar flow conditions in the SAXS region.

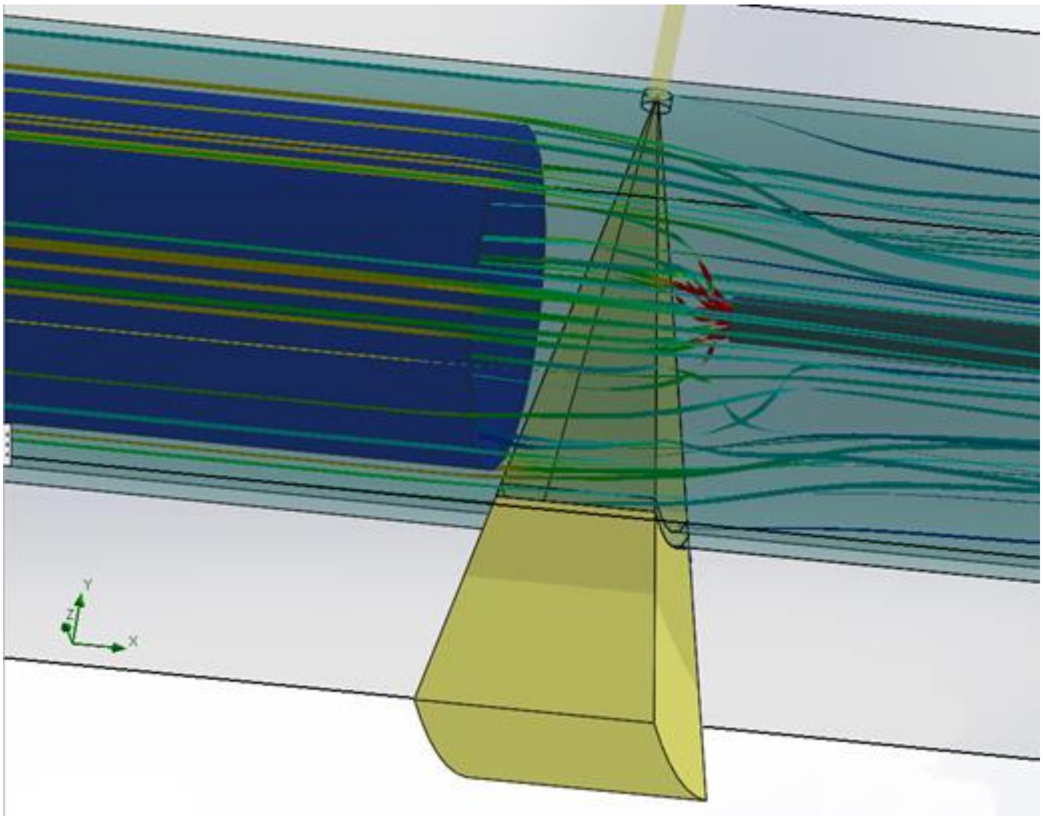

Supplementary Figure 2. **Flow simulation of the flow tube setup with sheath air system.** The dark blue tube is the inner flow tube and the light blue tube the outer flow tube for the sheath air. The gray tube is the core sampling for the DMPS and CPC. The yellow cone represents the x-ray beam and the maximal scattering angle (θ_{max}~15°) of the x-rays. Inside this cone the stream lines are stable and laminar for the SAXS measurements.

The integrated intensity profile derived with the differential background subtraction (DBS) for air is shown in Supplementary Figure 3. The integrated intensity cycle matches quite well with the relative particle concentration in the flow tube. The intensity of the resulting scattering curves is unfortunately too low for quantitative analysis.

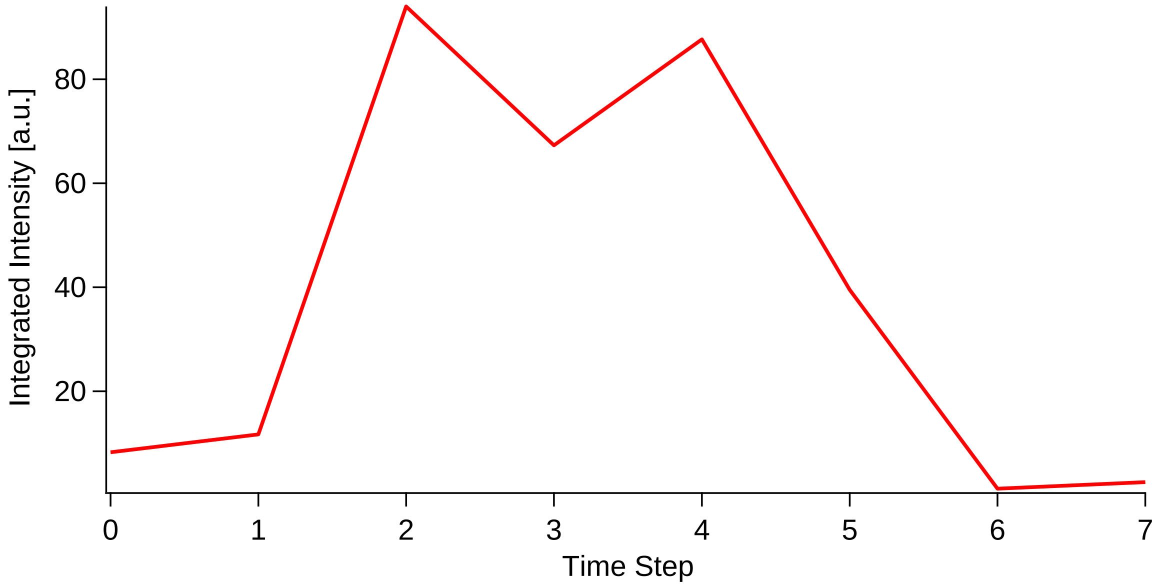

Supplementary Figure 3. **Integrated SAXS intensities of a DBS switching cycle in air at Elettra**. The time steps refer to the time steps of the switching module in the main text. The integrated intensity corresponds to the particle concentration in the SAXS beam. The shape of the curve matches with the switching cycle.

As described in the main text, helium was used to lower the background more than one order of magnitude. Thereby, the signal of the particles comes more in the foreground, which can be clearly seen in Supplementary Figure 4. It is the same type of graph as in Supplementary Figure 3, but with helium as carrier gas. The DBS switching cycle is more defined as in air. This agrees well with the lowering of the background by helium.

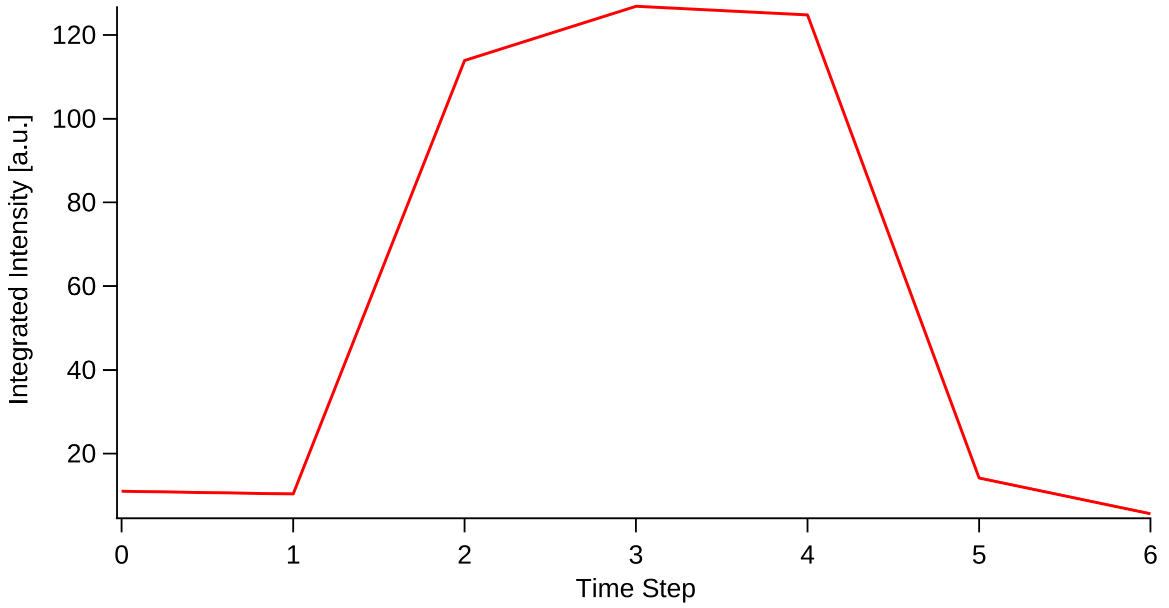

Supplementary Figure 4. **Integrated SAXS intensities of a DBS switching cycle in helium at Elettra**. The time steps refer to the time steps of the switching module in the main text. The integrated intensity corresponds to the particle concentration in the SAXS beam. The shape of the curve matches with the switching cycle.

Measurements taken at the ESRF show the advantage of a higher beam brilliance and beam stability. A detail of the integrated intensity time series is depicted in Supplementary Figure 5. There the signal was integrated between $q = 0.01 - 0.02$ Å^{-1}. In this context the stability of the background and signal of the particles during the DBS cycle are clearly visible. The average over several DBS cycles is shown in Supplementary Figure 6. The stability with the DBS system is therefore good enough to get reliable results from particles with ultra-low volume fraction.

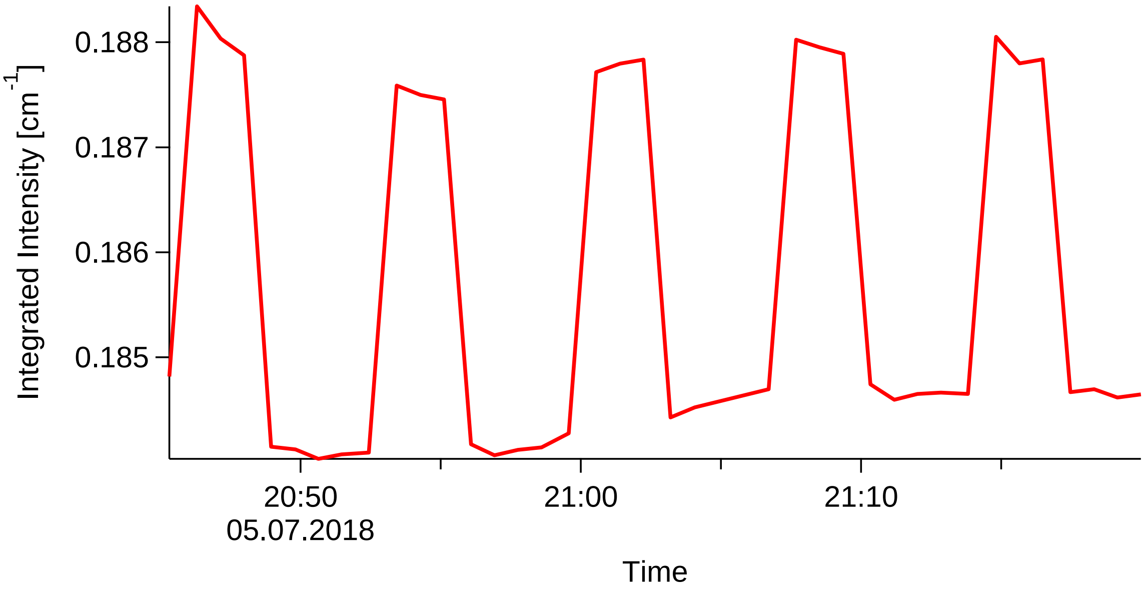

Supplementary Figure 5. **Detail of the integrated intensity over time with DBS switching in helium at the ESRF**. The integrated intensity is more stable at the ESRF than at ELETTRA. The switching is clearly visible and more pronounced than the small rise of the background.

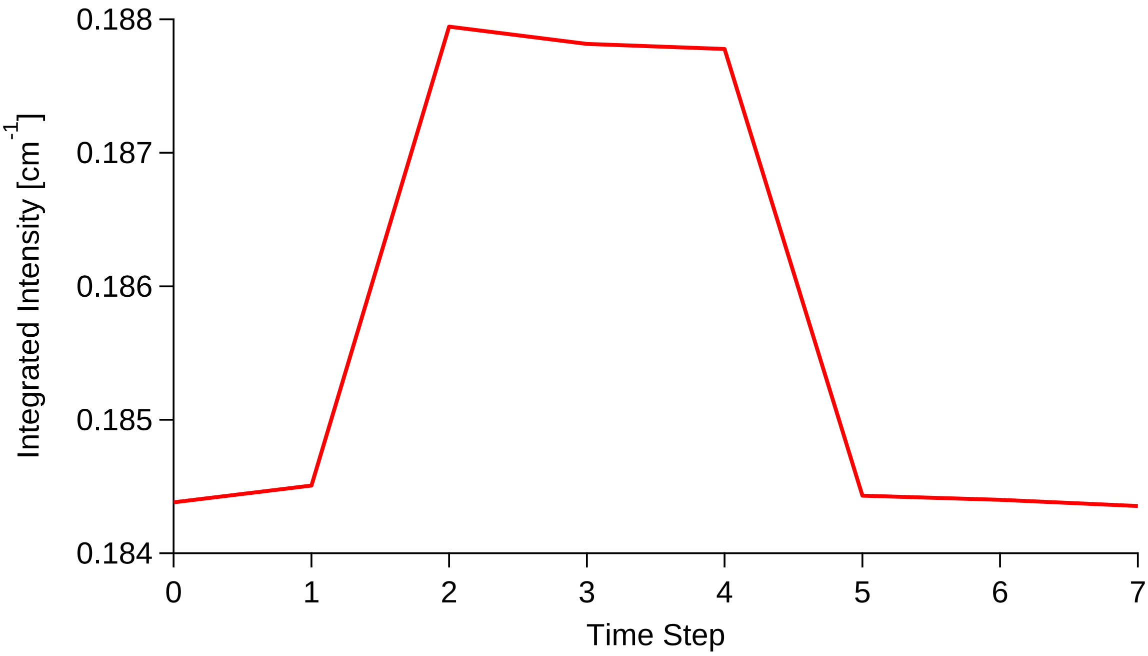

Supplementary Figure 6. **Integrated SAXS intensities of a DBS switching cycle in helium at the ESRF**. This results from averaging for every step of the DBS cycle of Supplementary Figure 5. The backgrounds before (step 0 and 1) and after (step 6 and 7) the particle measurements are very similar and can be considered as stable.
