# Supplementary material for: In-situ aerosol nanoparticle characterization by small angle X-ray scattering at ultra-low volume fraction
Source: Nat Commun. 2019 Mar 8;10:1122. doi: 10.1038/s41467-019-09066-4 (PMC6408461; doi:10.1038/s41467-019-09066-4)
Supplement: Supplementary file 2 — Peer Review File [file 41467_2019_9066_MOESM2_ESM.pdf]

Reviewers' comments:

Reviewer #1 (Remarks to the Author):

This paper reports on the analysis of very small aerosol nanoparticles by small-angle x-ray scattering (SAXS). A modification to the commonly used approach is presented to make the analysis possible in helium gas at ambient pressure for tungsten nanoparticles with number densities of ~1e6 per cc. This is an advancement over the previous studies, where the analysis was confined to low-pressure or vacuum conditions and a highly concentrated aerosol with a number density of ~1e12 per cc. The authors suggest that the future developments of the synchrotrone sources may allow the application of the proposed technique for in situ analysis of nanoparticles in the atmosphere.

This study is interesting, but its presentation has several crucial deficiencies. There are some problems with the assumptions made during analysis and some of the claims about the method's potential are not supported quantitatively. I do not recommend publishing this manuscript in the present form.

The paper is highly technical. In this study, the limits of the existing SAXS technique have been pushed to make the reported measurements possible. Something incredible and critical has been done, but the paper fails to tell the reader clearly, what those most critical elements are. The bottom half of the second page and the top half of the third page belong to methodology. That space could have been used more wisely, e.g., to outline the novelty and the essence of the approach, instead of wasting space on technical details.

A quantitative proof, at least some back-of-the-envelope calculations must have been presented to support claim that the technique utilized here can be extended to atmospheric conditions in the future. The authors admit that in this study 'the scattering contrast of … sample is close to the limit of detection'. To make measurements possible, the analysis was performed on the nanoparticles made of tungsten, which is a high-contrast material because of its large atomic mass. On the other hand, most of the atmospherically relevant nanoparticles, such as those produced during the atmospheric nucleation events, are made of light elements, such as carbon, oxygen, nitrogen, hydrogen, and sulfur. Next, this study has utilized helium as the carrier gas, which provides a significantly lower scattering background than the air. Finally, the number density of nanoparticles here was an order of magnitude higher than in the atmosphere, which is typically under 1e5 per cc (see next paragraph on the actual number density of the monomer nanoparticles, which I believe has been underestimated).

It is mentioned that the SAXS technique could not be used to derive the particle number density because of the low scattering contrast. The reported 1e6/cc comes from the scanning mobility measurements instead. However, based on the TEM microphotograph, the tungsten particles are aggregates, where each aggregate is made of 50-100 individual monomers. Hence, the monomer number density could be as high as 1e8/cc, rather than the 1e6/cc as reported by the differential mobility measurements.

Most probably, at such a high number density of the monomers, the aggregates were formed by coagulation immediately after tungsten vapor nucleation, rather than during charging / mobility analysis. This is very similar to the aggregates observed in many other studies utilizing graphite, silver, and tungsten as nanoparticle sources. Hence, SAXS measurements were probably done on the aggregates, which is fine, because in highly fractal aggregates, as shown in the TEM microphotograph, the monomers scatter the x-rays nearly independently of each other. Unless the method is sufficiently sensitive to recover the absolute scattered intensity accurately in order to retrieve the number density of the nanoparticles (and it is not), the scattered light wavelength dependence of individual monomers will not be very different from that of aggregates of monomers. I believe a relatively simple RDG or a more advanced T-Matrix calculation can be performed on a fractal aggregate of tungsten nanoparticles to support/refute this notion.

I see a great value in the capability of this technique to measure the size distribution of the monomers inside the aggregates. In all other aspects, this technique cannot compete with the existing sizing methods and I am not convinced that it can become an in situ method in foreseeable future, as claimed.

Reviewer #2 (Remarks to the Author):

Review of the article 'In-situ aerosol nanoparticle characterization by small angle x-ray scattering (SAXS) at ultra-low volume fraction' submitted to Nature Communications.

The authors present synchrotron Small Angle X-ray Scattering (SAXS) measurements of tungsten nanoparticles in an helium flow. The claim of this article is mainly that the aerosol is measured at a low concentration (C=106 particles per cm3). This concentration is compatible with environmental representative concentrations. The authors analyze the SAXS curves to extract the size distribution of the tungsten nanoparticles and compare the results with the results of a differential mobility particle sizer (DMPS).

Measuring such a low concentration of nanoparticles by SAXS is indeed a challenge especially at ambient atmospheric conditions. X-rays are scattered by electrons and one can estimate the scattering order of magnitude by something like I ~ C (ne b)^2 with C the particle concentration, b the Thomson factor (0.284 e-12 cm) and ne the number of electrons in the particles.

For a helium gaz at ambient pressure C= P/kT. If one considers tungsten particles of 2.7 nm with an electronic density of 3,7e24 cm-3 with C= 10^6 , this rough calculation gives an intensity more than 1000 times higher for the gaz electrons. This explains why previous authors have worked at concentrations of at least 10^10 cm-3 and highlight the challenge of this manuscript.

The authors clearly present the challenges to obtain a statistically relevant measurement (beam stability, particle deposition on windows, background level…). Several technical modifications have been performed to manage these difficulties. The use of Helium instead of air enable to reduce the background by a factor of ~ (7/2)^2 because of the reduction of the number of electrons. The Kapton windows have been covered by an aluminum layer to prevent electrostatic interactions, a special flow is used to reduce the deposition. It is surprising that the beam stop is not introduced inside the chamber to prevent the scattering from the second Kapton window. The authors have also developed a method to take into account the background. They inject the particles in sequence and measure a background before and after the aerosol measurement. The two backgrounds are then averaged. They call this process differential background subtraction (DBS). The measurement challenge is indeed interesting if clearly demonstrated but the requirement of a synchrotron beam also severely reduce the potential practical use of this method.

I have several concerns about the evidence that the measured signal is indeed due to the low concentration aerosol. If one tries to estimate the ratio of nanoparticles in the beam and on the windows, one gets something like ~C e r2 where r is the radius of the nanoparticle, e is the length of the scattering volume and C the NP concentration in this scattering volume. This is a very small number and it means that the precautions to have no contributions from deposited NP on the windows have to be very efficient.

In the SI documents, Figure 1 presents the integrated intensity obtained after several measurements. This demonstrates a significant deposition of NP on the kapton windows.
Unfortunately, to convince the reader that the precautions taken are successful, the authors do not

display the same figure as the Figure 1, but instead, they apply the DBS method to produce Figure 4 of the SI. Even if the modifications enabled to reduce the number of deposited particles, a small amount would give a significant signal. If like in Figure 1, it exists a slope in the deposition curve, the DBS method may not be able to correct this contribution.

To convince the reader, the authors should show the equivalent of Figure 1 without the DBS subtraction to appreciate the efficiency of the deposition reduction. It would also be very important to show the scattering curves of the backgrounds before and after the particle injection and the scattering curves obtain while the nanoparticles are present. This would enable to estimate the amount of deposition and the importance of the signal coming from the aerosol. This figure could replace figure 3 which is not very useful and should be in the SI.

Another very important point to me is the presentation of the intensity in arbitrary units. The authors say "Due to the low particle concentration for SAXS, we were not able to calibrate our SAXS curves to an absolute scale, which is necessary for calculating the number concentration."

It would be necessary to explain why. The authors are using a synchrotron beam which is precisely monitored and a Pilatus detector which is having an efficiency close to 1 for photon at 8 keV. They have calibrated the sample detector distance, they know the measurement time and the pixel size etc… So everything needed to have a measurement in absolute intensity (cm-1) is available. Performing this intensity calibration would add a very strong argument to the authors. Indeed, if only the aerosol particles contribute, the obtained intensity should match the expected one for 10^6 particle/cm3 having a scattering length density of tungsten (~1.03 10^12 cm-2) measured in helium in STP (~1.5 10^7 cm-2). For dispersed aerosols with radius r=2.7 nm intensities as low as 10-8 cm-1 are expected. If the aerosol are agglomerated the intensity will increase. The value of the experimental absolute intensity is needed to see if the data are in the expected range. This discussion is missing and it is far more important than the one on the size distribution.

I will not go into other details. To me, the manuscript in the present form do not meet the nature comm. requirement to fully demonstrate the principal claim in a convincing manner. The manuscript should not be published in the present form. If significant improvements in the presentation of the data analysis and calibration is added the manuscript, it could perhaps be resubmitted.

Reviewer #3 (Remarks to the Author):

Comments on "In-situ aerosol nanoparticle characterization by small angle x-ray scattering (SAXS) at ultra-low volume fraction" by Bauer et al.

Atmospheric aerosol particles have been intensively studied due to their impact on air pollution and climate change. Current measurement techniques (such as Scanning Mobility Particle Sizers, SMPS) are subject to uncertainties, because (1) the equilibrium charge distribution of small nanoparticles, the basis of SMPS measurement, hasn't be experimentally validated; (2) it is not clear if properties of aerosols will change in the measuring devices (e.g., activation chamber), a different environment compared to real atmosphere.

Bauer et al. developed an in-situ measurement technique SAXS for measuring the size distribution of atmosphere aerosols. Using helium as carrier gas, they are able to determine the aerosol size distribution with concentrations down to 10**6 cm-3, close to the ambient condition. The experiments with tungsten particles show that this new technique is able to detect very small primary nanoparticles of 5 nm, while conventional SMPS shows a mean diameter of ~30 nm. The differences have been attributed to the formation of agglomerates during the charging process of SMPS.

Some of the authors on the paper have a reputation for publishing very high profile, well regarded,

and well-cited papers in leading journals (including Science), which help explain the nucleation event of atmospheric nanoparticles. This paper should have a similar impact in the atmospheric science community if the authors could clarify the following questions:

(1) Carrier gas and detection limit

The motivation of this development is to perform in-situ measurement of aerosol nanoparticles under real atmospheric conditions. But helium is used as carrier gas instead of ambient air. This is not a real atmospheric condition and the authors need to provide some justifications. For example, are aerosol formation/transformation in helium the same or similar as that in air? If so, lab experiments in helium can be used to simulate the formation of aerosols in ambient air.

The authors have tested this technique under aerosol concentrations of 10**6 cm-3. In real atmosphere, aerosol concentrations are mostly below 10**5 cm-3, close to the background noise. The authors need to include some discussions how this gap can be bridged.

(2) Absolute concentration

The authors suggested that they were able to get the right shape of aerosol size distribution but not the absolute concentration due to the low concentration. Is there a threshold value, above which the calculation of concentration become possible? In the aerosol nucleation studies, the absolute concentration is needed to derive the nucleation rate and to test different formation mechanisms. If this new technique cannot provide such information, the authors need to explain how an accurate shape of aerosol size distribution can be important for aerosol formation studies.

(3) Difference between SAXS and DMPS/SMPS
For me, the difference between SAXS and DMPS may be caused by the follow reasons: they were not measuring the same aerosol sample; or they were measuring the same sample and at least one method is not working.

The authors attributed the difference to the first reason, because they think that no agglomerate was formed "In our case the SAXS curve indicates that there is only one structural level necessary to fit the data, since there is no second Porod regime visible in the lower q-range".

I am not an expert on SAXS, but I wonder what kind of scattering signals will the SAXS produce for a chain-style agglomerate, e.g., if the agglomerate is formed by a few connected primary particles, will the scattering signal looks more like an intensified signal from primary particles, or a signal from a larger (double- or triple-sized) particle. If the agglomerates come from the charging process, the authors should see the same picture if they also charge aerosol samples for SAXS measurement. Have you done such experiments? Because it will strongly consolidate the conclusions.

Authors Response to the Reviewer Comments regarding the manuscript “In-situ aerosol nanoparticle characterization by small angle x-ray scattering (SAXS) at ultra-low volume fraction” by Bauer et al.

Response to Reviewer #1:

This paper reports on the analysis of very small aerosol nanoparticles by small-angle x-ray scattering (SAXS). A modification to the commonly used approach is presented to make the analysis possible in helium gas at ambient pressure for tungsten nanoparticles with number densities of ~1e6 per cc. This is an advancement over the previous studies, where the analysis was confined to low-pressure or vacuum conditions and a highly concentrated aerosol with a number density of ~1e12 per cc. The authors suggest that the future developments of the synchrotrone sources may allow the application of the proposed technique for in situ analysis of nanoparticles in the atmosphere.

This study is interesting, but its presentation has several crucial deficiencies. There are some problems with the assumptions made during analysis and some of the claims about the method's potential are not supported quantitatively. I do not recommend publishing this manuscript in the present form.

The paper is highly technical. In this study, the limits of the existing SAXS technique have been pushed to make the reported measurements possible. Something incredible and critical has been done, but the paper fails to tell the reader clearly, what those most critical elements are. The bottom half of the second page and the top half of the third page belong to methodology. That space could have been used more wisely, e.g., to outline the novelty and the essence of the approach, instead of wasting space on technical details.
We want to thank reviewer 1 for the constructive feedback. We have moved the text passage mentioned by the reviewer to the methodology part and tried to be more specific on the essence of the approach namely to minimize the effects of the background to get good statistics. As will be shown below and in the manuscript we have now much better data that do support our claims.

A quantitative proof, at least some back-of-the-envelope calculations must have been presented to support claim that the technique utilized here can be extended to atmospheric conditions in the future. The authors admit that in this study ‘the scattering contrast of ... sample is close to the limit of detection’. To make measurements possible, the analysis was performed on the nanoparticles made of tungsten, which is a high-contrast material because of its large atomic mass. On the other hand, most of the atmospherically relevant nanoparticles, such as those produced during the atmospheric nucleation events, are made of light elements, such as carbon, oxygen, nitrogen, hydrogen, and sulfur. Next, this study has utilized helium as the carrier gas, which provides a significantly lower scattering background than the air. Finally, the number density of nanoparticles here was an order of magnitude higher than in the atmosphere, which is typically under 1e5 per cc (see next paragraph on the actual number density of the monomer nanoparticles, which I believe has been underestimated).

It is mentioned that the SAXS technique could not be used to derive the particle number density because of the low scattering contrast. The reported 1e6/cc comes from the scanning mobility measurements instead. However, based on the TEM microphotograph, the tungsten particles are aggregates, where each aggregate is made of 50-100 individual monomers. Hence, the monomer number density could be as high as 1e8/cc, rather than the 1e6/cc as reported by the differential mobility measurements.
The concerns of reviewer 1 are well justified. The limit of detection in the previous manuscript version though has been determined by the comparatively low photon flux at the SAXS beamline at Elettra. Still, these measurements and preparation work were crucial to optimize our system. Our latest

experiments at ESRF demonstrate the feasibility of our approach and provide detailed insight to the particle properties. Remarkably, the estimate of monomers per aggregate by the reviewer covers exactly the range which we have now determined experimentally. The number density of slightly above 10^6 /cc was measured with a DMPS and is therefore a measurement for the number of aggregates which form right after the particle generator. Our conclusions have therefore been amended accordingly.

Most probably, at such a high number density of the monomers, the aggregates were formed by coagulation immediately after tungsten vapor nucleation, rather than during charging / mobility analysis. This is very similar to the aggregates observed in many other studies utilizing graphite, silver, and tungsten as nanoparticle sources. Hence, SAXS measurements were probably done on the aggregates, which is fine, because in highly fractal aggregates, as shown in the TEM microphotograph, the monomers scatter the x-rays nearly independently of each other. Unless the method is sufficiently sensitive to recover the absolute scattered intensity accurately in order to retrieve the number density of the nanoparticles (and it is not), the scattered light wavelength dependence of individual monomers will not be very different from that of aggregates of monomers. I believe a relatively simple RDG or a more advanced T-Matrix calculation can be performed on a fractal aggregate of tungsten nanoparticles to support/refute this notion.
Reviewer 1 anticipated exactly what we have measured now. As mentioned above the aggregates are formed immediately after the generator. With the new SAXS measurements taken at the ERSF we were able to resolve the aggregates and primary particles and the fractal dimension. We have performed an absolute calibration with water which allowed us to retrieve the volume fraction of the particles. In our case the volume fraction is in the order of 10^{-10}. Our new results show how important a stable and high intensity beam source is for our experiment.

I see a great value in the capability of this technique to measure the size distribution of the monomers inside the aggregates. In all other aspects, this technique cannot compete with the existing sizing methods and I am not convinced that it can become an in situ method in foreseeable future, as claimed.
We do not see our approach possibly replacing common aerosol techniques. However, every technique has its own value and gives different information on the nanoparticles. We see SAXS as an additional method, which can give *in-situ* information on the primary particles and the aggregates as shown now in the results. Therefore this technique provides significant new insight to aerosol nanoparticle properties. We even have indications now that our method works also with air which will be a decisive step forward and further increases the value of our approach.

Response to Reviewer #2:

Review of the article 'In-situ aerosol nanoparticle characterization by small angle x-ray scattering (SAXS) at ultra-low volume fraction' submitted to Nature Communications.

The authors present synchrotron Small Angle X-ray Scattering (SAXS) measurements of tungsten nanoparticles in an helium flow. The claim of this article is mainly that the aerosol is measured at a low concentration (C=106 particles per cm3). This concentration is compatible with environmental representative concentrations. The authors analyze the SAXS curves to extract the size distribution of the tungsten nanoparticles and compare the results with the results of a differential mobility particle sizer (DMPS).

Measuring such a low concentration of nanoparticles by SAXS is indeed a challenge especially at ambient atmospheric conditions. X-rays are scattered by electrons and one can estimate the scattering order of magnitude by something like I ~ C (ne b)^2 with C the particle concentration, b the Thomson factor (0.284 e-12 cm) and ne the number of electrons in the particles.

For a helium gaz at ambient pressure C= P/kT. If one considers tungsten particles of 2.7 nm with an electronic density of 3,7e24 cm-3 with C= 10^6 , this rough calculation gives an intensity more than 1000 times higher for the gaz electrons. This explains why previous authors have worked at concentrations of at least 10^10 cm-3 and highlight the challenge of this manuscript.

The authors clearly present the challenges to obtain a statistically relevant measurement (beam stability, particle deposition on windows, background level…). Several technical modifications have been performed to manage these difficulties. The use of Helium instead of air enable to reduce the background by a factor of ~ (7/2)^2 because of the reduction of the number of electrons. The Kapton windows have been covered by an aluminum layer to prevent electrostatic interactions, a special flow is used to reduce the deposition. It is surprising that the beam stop is not introduced inside the chamber to prevent the scattering from the second Kapton window. The authors have also developed a method to take into account the background. They inject the particles in sequence and measure a background before and after the aerosol measurement. The two backgrounds are then averaged. They call this process differential background subtraction (DBS). The measurement challenge is indeed interesting if clearly demonstrated but the requirement of a synchrotron beam also severely reduce the potential practical use of this method.

We appreciate the careful thoughts of reviewer 2 and his recognition of challenges in our experiment.

We would like to point out though that the ratio of the Scattering Length Density (SLD) from air to helium is about 15/2 rather than 7/2 since air mostly consists out of diatomic molecule and helium is monoatomic. This ratio is now also mentioned in the text for clarification.

Regarding the beam stop we thought about putting it inside the flow tube, but there were several issues encountered. First, since the beam stop would be very close to the scattering region it would restrict the scattering angle range in the low q range drastically. Second, a diode is installed on the beam stop in front of the detector to measure the transmission, which is very important for the absolute calibration. These are the main reasons, why we haven't followed this idea further.

I have several concerns about the evidence that the measured signal is indeed due to the low concentration aerosol. If one tries to estimate the ratio of nanoparticles in the beam and on the windows, one gets something like ~C e r2 where r is the radius of the nanoparticle, e is the length of the scattering volume and C the NP concentration in this scattering volume. This is a very small number and it means that the precautions to have no contributions from deposited NP on the windows have to be very efficient.
In the SI documents, Figure 1 presents the integrated intensity obtained after several measurements. This demonstrates a significant deposition of NP on the kapton windows.
Unfortunately, to convince the reader that the precautions taken are successful, the authors do not display the same figure as the Figure 1, but instead, they apply the DBS method to produce Figure 4 of the SI. Even if the modifications enabled to reduce the number of deposited particles, a small amount would give a significant signal. If like in Figure 1, it exists a slope in the deposition curve, the DBS method may not be able to correct this contribution.
We agree with the reviewer, that deposition on the windows and scattering of the windows is indeed one of the biggest issues, which we have now addressed in our manuscript in more detail. Figure 1 of the SI documents is one of our first measurements at the first measurement campaign at ELETTRA. There we realized that deposition needs to be properly accounted for. Therefore, we wanted to show this graph in the SI to the advanced reader, who wants to be aware of all the background effects we have identified during our development phase.

To convince the reader that the background is stable enough, we have now included in the supplementary information in Fig. 6 a detail of the integrated intensity of the SAXS measurements at ESRF. There, only a small rise of the background is visible compared to the signal during the particle phase of the DBS. The average of each step of the DBS is shown in Fig. 7 in the supplementary

information. If there would be a strong increase between the background before and after the particles the average would show this as well, which is not the case. Therefore, we are sure that we have resolved most background issues with our current method.

To convince the reader, the authors should show the equivalent of Figure 1 without the DBS subtraction to appreciate the efficiency of the deposition reduction. It would also be very important to show the scattering curves of the backgrounds before and after the particle injection and the scattering curves obtain while the nanoparticles are present. This would enable to estimate the amount of deposition and the importance of the signal coming from the aerosol. This figure could replace figure 3 which is not very useful and should be in the SI.

See our response above. Since the scattering of the background at ESRF is about 1 cm^{-1} the signal added by the particles is only 10^{-3} cm^{-1} at the lower q values, the difference between the scattering curves is barely visible. Therefore, we integrate the scattering intensity to make this small difference visible, which is shown in the SI.

We have chosen Fig. 3 particularly for the main text, to point out the difference in scattering from air and helium. All the other SAXS curves taken in helium (even with particles in the flow tube) look very similar to the helium SAXS curve of Figure 3 since the primary scattering effect (background) is coming from the window material and the gas. Only by statistical evaluation of these curves with particles on/off the signal is finally retrieved. We therefore consider this figure essential for the visualization of background effects.

Another very important point to me is the presentation of the intensity in arbitrary units. The authors say "Due to the low particle concentration for SAXS, we were not able to calibrate our SAXS curves to an absolute scale, which is necessary for calculating the number concentration."

It would be necessary to explain why. The authors are using a synchrotron beam which is precisely monitored and a Pilatus detector which is having an efficiency close to 1 for photon at 8 keV. They have calibrated the sample detector distance, they know the measurement time and the pixel size etc… So everything needed to have a measurement in absolute intensity (cm-1) is available. Performing this intensity calibration would add a very strong argument to the authors. Indeed, if only the aerosol particles contribute, the obtained intensity should match the expected one for 10^6 particle/cm3 having a scattering length density of tungsten (~1.03 10^12 cm-2) measured in helium in STP (~1.5 10^7 cm-2). For dispersed aerosols with radius r=2.7 nm intensities as low as 10-8 cm-1 are expected. If the aerosol are agglomerated the intensity will increase. The value of the experimental absolute intensity is needed to see if the data are in the expected range. This discussion is missing and it is far more important than the one on the size distribution.

As mentioned by the reviewer, a precisely monitored beam is necessary for absolute calibration. Our new measurements at the ESRF were calibrated to an absolute scale since the beam was monitored more precisely and was more stable than at ELETTRA. At ELETTRA we took out the ionization chamber to connect the flow tube directly to the vacuum of the beam line. The diode at the beam stop was not sensitive enough at the ELETTRA for our small changes arising from the relatively low concentration of particles. Therefore, it was not possible for us to get an absolute calibration at ELETTRA, even with a PILATUS detector, but the new measurements from ESRF are now absolute calibrated.

I will not go into other details. To me, the manuscript in the present form do not meet the nature comm. requirement to fully demonstrate the principal claim in a convincing manner. The manuscript should not be published in the present form. If significant improvements in the presentation of the data analysis and calibration is added the manuscript, it could perhaps be resubmitted.

We hope that we have now addressed all major concerns and improved the calibration and analysis to satisfy the points raised.

Responds to Reviewer #3:

Atmospheric aerosol particles have been intensively studied due to their impact on air pollution and climate change. Current measurement techniques (such as Scanning Mobility Particle Sizers, SMPS) are subject to uncertainties, because (1) the equilibrium charge distribution of small nanoparticles, the basis of SMPS measurement, hasn't be experimentally validated; (2) it is not clear if properties of aerosols will change in the measuring devices (e.g., activation chamber), a different environment compared to real atmosphere.

*Bauer et al. developed an in-situ measurement technique SAXS for measuring the size distribution of atmosphere aerosols. Using helium as carrier gas, they are able to determine the aerosol size distribution with concentrations down to 10**6 cm-3, close to the ambient condition. The experiments with tungsten particles show that this new technique is able to detect very small primary nanoparticles of 5 nm, while conventional SMPS shows a mean diameter of ~30 nm. The differences have been attributed to the formation of agglomerates during the charging process of SMPS.*

Some of the authors on the paper have a reputation for publishing very high profile, well regarded, and well-cited papers in leading journals (including Science), which help explain the nucleation event of atmospheric nanoparticles. This paper should have a similar impact in the atmospheric science community if the authors could clarify the following questions:

(1) Carrier gas and detection limit

The motivation of this development is to perform in-situ measurement of aerosol nanoparticles under real atmospheric conditions. But helium is used as carrier gas instead of ambient air. This is not a real atmospheric condition and the authors need to provide some justifications. For example, are aerosol formation/transformation in helium the same or similar as that in air? If so, lab experiments in helium can be used to simulate the formation of aerosols in ambient air.
Our original plan was to study new particle formation using SAXS. However, we soon realized that these experiments are more demanding and need thorough development. Replacing air by helium was an important step in achieving signals and understanding the behavior of our system. We do not claim that the formation of aerosols is the same in helium and air, but we can measure the particles similar to several studies that have already used helium, e.g., Maißner, A. et al. (2015) (cited also in the text), Brus, D. et al. The Journal of Chemical Physics 122, (2005) or early studies as Ahn, K.-H. & Liu, B.Y.H. Journal of Aerosol Science 21, 263 (1990). In fact, these studies served as role models where we found hints and tips how to operate our aerosol instruments with helium. Importantly, we are now able to measure the particles with DMPS *and* SAXS in helium and the next step is to adapt it for air. In order to avoid misunderstanding we have reduced emphasizing atmospheric relevance. “Ambient conditions” solely refers to the fact that we have operated our system at ambient pressure and room temperature.

*The authors have tested this technique under aerosol concentrations of 10**6 cm-3. In real atmosphere, aerosol concentrations are mostly below 10**5 cm-3, close to the background noise. The authors need to include some discussions how this gap can be bridged.*
We agree that our settings are not immediately representative for atmospheric conditions. However, concentrations of 10^6 /cc may occur in the vicinity of atmospheric particle sources. If feasible in air our approach may thus provide insight to the formation of particles (given that the source can be brought to a synchrotron). It should be noted though that for SAXS $10^6/cc$ is an ultra-low concentration due to the resulting low volume fraction as described in the text. On the other hand for aerosol instruments it is at the higher end of the measurement range. In this study we wanted to bridge the gap between the methods. New window materials and a very stable beam and background are necessary to go to lower concentrations as mentioned also in the text. Based on our recent experience

at ESRF we are optimistic that this method can be applied to “real” aerosols using air as carrier gas. The lower resolvable concentration range needs to be tested though.

(2) Absolute concentration

The authors suggested that they were able to get the right shape of aerosol size distribution but not the absolute concentration due to the low concentration. Is there a threshold value, above which the calculation of concentration become possible? In the aerosol nucleation studies, the absolute concentration is needed to derive the nucleation rate and to test different formation mechanisms. If this new technique cannot provide such information, the authors need to explain how an accurate shape of aerosol size distribution can be important for aerosol formation studies.

With the new measurements done at ESRF we were able to do an absolute calibration. Thereby we obtained absolute concentration values. At ELETTRA an absolute calibration was not possible for several reasons as explained in our reply to referee 2. With the new data we could get information on the primary particles as well on the agglomerates.

(3) Difference between SAXS and DMPS/SMPS

For me, the difference between SAXS and DMPS may be caused by the follow reasons: they were not measuring the same aerosol sample; or they were measuring the same sample and at least one method is not working.

As the new measurements show SAXS and DMPS are in good agreement suggesting that both instruments are measuring the same sample. The former ELETTRA results were not sensitive enough to resolve the aggregates (supporting the guess of referee 3).

The authors attributed the difference to the first reason, because they think that no agglomerate was formed “In our case the SAXS curve indicates that there is only one structural level necessary to fit the data, since there is no second Porod regime visible in the lower q-range”.

I am not an expert on SAXS, but I wonder what kind of scattering signals will the SAXS produce for a chain-style agglomerate, e.g., if the agglomerate is formed by a few connected primary particles, will the scattering signal looks more like an intensified signal from primary particles, or a signal from a larger (double- or triple-sized) particle. If the agglomerates come from the charging process, the authors should see the same picture if they also charge aerosol samples for SAXS measurement. Have you done such experiments? Because it will strongly consolidate the conclusions.

The extended q-range together with much higher beam intensity available at ESRF allowed us to resolve this issue in great detail. We hope the new results can show the strength of SAXS to measure the primary particles as well as the aggregates. As mentioned above, unfortunately we haven’t seen the second Porod level on our previous experiments due to the comparatively weak beam conditions at ELETTRA.

Reviewers' comments:

Reviewer #1 (Remarks to the Author):

Overall, I am satisfied with the revised version. However, before the manuscript can be published, one issue must be resolved:

In the abstract and throughout the manuscript, a clear distinction between spherical tungsten monomers and their aggregates must be made. For instance, the 10^6 /cc number concentration given in the abstract is highly misleading, as it is the concentration of aggregates, not the concentration of tungsten spheres. The actual concentration of tungsten spheres would be a factor of ~100 higher, i.e., 10^8 /cc.

Reviewer #2 (Remarks to the Author):

The experiments performed at ESRF enable a proper calibration of the scattered intensity (1/cm) and are more convincing regarding the control of particle adsorption at the windows.
However, i wonder why the authors choose to change the representation of the background effect. It is presented as a serie of averaged backgrounds in fig 1 of the SI which clearly display a slope. It is not shown in this way for the new experiments. Maybe a direct comparison on a similar figure with probably two y axis could be clearer. In their answer to our questions, the authors mentioned that the background level is of about 1cm^-1. However, in figure 3, the background is still shown in arbitrary unit. Is it because this correspond to measurements performed at Elletra? Is it because no measurements in air have been performed in ESRF?

The SAXS results are analyzed using the so-called Beaucage model. But the terminology of the parameters is not correct. As stated by the authors, a mass fractal will display a I(q)~q^(-df) in a q range corresponding to distances larger than the primary particle and smaller than the aggregate size. Above q~1/R_1, the scattering enters the Porod regime where scattering is controlled by the transition between scattering length density (SLD) of the particle and the surrounding media. The observed slope of -4 corresponds to a sharp transition and the exponent should not be called fractal and noted df. It is possible to observe scattering from fractal surfaces but in this case the slope is no longer -4.

It is also puzzling that the authors do not used the DMPS measurements to further explain the scattering curves. There is indeed an agreement between the volume fractions obtained between the two techniques. But the volume fraction is obtained from SAXS using the computation of the invariant. The invariant is a global number which is independent of the structure of the sample. It just depends on the SLD and the volume fraction. No comparison is performed on the size distribution of the aggregates. The DMPS measurements show a really broad distribution. If this size distribution could be taken into account in the SAXS analysis, what would be the impact on the fractal dimension of the aggregates? Isn’t it a link between the broadness of the distribution and the fractal dimension of the aggregates?

I think the SAXS analysis should be reworked to employ proper notations and could compare more precisely the two techniques SAXS and DMPS. Especially because the author claim in the introduction that information on the structure and size distribution of the aggregates is available.

Finally, I have interrogation on the introduction and general claim of the article. The introduction starts by pointing the problem of the need to “remove the particle from their original environment” and conclude that the measurements of ultra-low volume fraction is possible. This would make people think that the proposed technique could be usable for aerosol science in situ in ambient pressure for

their own topics. But the limitations of the presented technique are rather severe. It works in an Helium atmosphere and requires the use of a synchrotron light source! This would only be available for a few high-end research activities in aerosol science in Helium atmosphere. I think the introduction should be reworked to also clearly state these severe limitations.

Reviewer #3 (Remarks to the Author):

The authors have carried out additional experiments which helped to answer my early questions. I am satisfied and would recommend its publication in Nature Communications.

Reviewer #1 (Remarks to the Author):

Overall, I am satisfied with the revised version. However, before the manuscript can be published, one issue must be resolved:

In the abstract and throughout the manuscript, a clear distinction between spherical tungsten monomers and their aggregates must be made. For instance, the 10^6 /cc number concentration given in the abstract is highly misleading, as it is the concentration of aggregates, not the concentration of tungsten spheres. The actual concentration of tungsten spheres would be a factor of ~100 higher, i.e., 10^8 /cc.

Reply: We understand that the phrase “tungsten blue oxide nanoparticles” in the abstract might be misleading and can be understood as aggregates or primary particles, respectively. In order to avoid confusion we therefore replaced “tungsten blue oxide nanoparticles” by “aerosol nanoparticles”. This should make clear that the mentioned concentration of 10^6 /cc simply refers to the number concentration of particles as determined from aerosol instrumentation, no matter what the structure/composition of the particles is. The second mentioning of this appears on page 5, line 5, where it is clearly stated that it is the aerosol nanoparticle concentration in the range of 10^6/cc. Hopefully this resolves the concerns of Reviewer 1.

Reviewer #2 (Remarks to the Author):

The experiments performed at ESRF enable a proper calibration of the scattered intensity (1/cm) and are more convincing regarding the control of particle adsorption at the windows.
However, i wonder why the authors choose to change the representation of the background effect. It is presented as a serie of averaged backgrounds in fig 1 of the SI which clearly display a slope. It is not shown in this way for the new experiments. Maybe a direct comparison on a similar figure with probably two y axis could be clearer. In their answer to our questions, the authors mentioned that the background level is of about 1cm^-1. However, in figure 3, the background is still shown in arbitrary unit. Is it because this correspond to measurements performed at Elletra? Is it because no measurements in air have been performed in ESRF?

Reply: As stated in the beginning of the SI this information is intended to illustrate pitfalls and progress in these studies. Fig. 1 of the SI shows the very first data taken at Elettra where the rising background due to deposition is manifested. After several changes to our system and the use of the DBS method the background does not increase anymore as can be seen from Fig. 5 in the SI where we show data from ESRF with the intensity scale in units of [cm^(-1)]. Fig. 3 of the manuscript indeed shows data from Elettra. Even though we do not have absolute values available for these data, the background reduction by one order of magnitude due to changing from air to helium clearly becomes visible. Such a figure is not available from ESRF data as we had a slightly different set-up with the flow tube not immediately connected to the vacuum line of the beam. So some scattering from air is superimposed in the ESRF signals. Still, we obtained these improved results. By the way, this “artifact” greatly increased our optimism that SAXS will be applicable to similar measurements in air.

The SAXS results are analyzed using the so-called Beaucage model. But the terminology of the parameters is not correct. As stated by the authors, a mass fractal will display a I(q)~q^(-df) in a q range corresponding to distances larger than the primary particle and smaller than the aggregate size. Above q~1/R_1, the scattering enters the Porod regime where scattering is controlled by the transition between scattering length density (SLD) of the particle and the surrounding media. The observed slope of -4 corresponds to a sharp transition and the exponent should not be called fractal and noted df. It is possible to observe scattering from fractal surfaces but in this case the slope is no longer -4.

Reply: We have now reworked parts of the Method section to employ proper notation. In this context, we relabeled the power-law regime in the method section and made a clear distinction between the power of -4 (Porod regime) and the fractal-dimension d_f. Hopefully this resolves the concerns of Reviewer 2.

It is also puzzling that the authors do not used the DMPS measurements to further explain the scattering curves. There is indeed an agreement between the volume fractions obtained between the two techniques. But the volume fraction is obtained from SAXS using the computation of the invariant. The invariant is a global number which is independent of the structure of the sample. It just depends on the SLD and the volume fraction. No comparison is performed on the size distribution of the aggregates. The DMPS measurements show a really broad distribution. If this size distribution could be taken into account in the SAXS analysis, what would be the impact on the fractal dimension of the aggregates? Isn't it a link between the broadness of the distribution and the fractal dimension of the aggregates?
I think the SAXS analysis should be reworked to employ proper notations and could compare more precisely the two techniques SAXS and DMPS. Especially because the author claim in the introduction that information on the structure and size distribution of the aggregates is available.

Reply: We have tried several approaches to get relevant information out of the SAXS curve. Every approach revealed almost the same results about the primary particles and the fractal dimension of the aggregates, which emphasizes the robustness of the results. The Beaucage model was chosen for presentation, since it is the easiest to retrieve further information out of the fit-parameters.

The use of the DMPS distribution to construct a SAXS curve is not very useful, since SAXS sees the primary particles and aggregates whereas the DMPS system sees only the aggregates. This is as well discussed in the paper in the paragraph where DMPS and SAXS is compared. However, we gain a lot of information by combining these two instruments. From SAXS we get the structure (fractal dimension, reflecting the type of aggregation phenomena) and size of the primary particles and from the DMPS we get the size distribution of the aggregates! This is as well stated in the conclusions. Linking the fractal dimension to the broadness of the distribution is not straight forward and likely leads to erroneous results for log-normal distributions as has been shown by Sorensen, C. M. & Wang, G. M. *Size distribution effect on the power law regime of the structure factor of fractal aggregates*. Phys. Rev. E 60, 7143–7148 (1999). We therefore consider this request of reviewer 2 beyond the scope of our manuscript.

Finally, I have interrogation on the introduction and general claim of the article. The introduction starts by pointing the problem of the need to “remove the particle from their original environment” and conclude that the measurements of ultra-low volume fraction is possible. This would make people think that the proposed technique could be usable for aerosol science in situ in ambient pressure for their own topics. But the limitations of the presented technique are rather severe. It works in an Helium atmosphere and requires the use of a synchrotron light source! This would only be available for a few high-end research activities in aerosol science in Helium atmosphere. I think the introduction should be reworked to also clearly state these severe limitations.

Reply: In the introduction we say that new ways of nanoparticle analysis are desirable which in our opinion is a quite general statement. It should be noted that the last sentence of the abstract clearly states that the latest generation synchrotrons is needed in order to perform these kinds of experiments. To make this clearer we have added a short phrase at the end of the concluding section where this has been actually addressed.

Reviewer #3 (Remarks to the Author):

The authors have carried out additional experiments which helped to answer my early questions. I am satisfied and would recommend its publication in Nature Communications.

Reply: We certainly appreciate the positive comment by Referee 3.

REVIEWERS' COMMENTS:

Reviewer #2 (Remarks to the Author):

The corrections about the Porod regime have been performed and my questions have been answered.
I have no other questions.
I still think that the abstract could have more clearly stated that the proposed technique will not be available for classical aerosol research or analysis.
The requirement of a synchrotron radiation severely limits this technique too a few high end research activities.

REVIEWERS' COMMENTS:

Reviewer #2 (Remarks to the Author):

The corrections about the Porod regime have been performed and my questions have been answered. I have no other questions.
I still think that the abstract could have more clearly stated that the proposed technique will not be available for classical aerosol research or analysis.
The requirement of a synchrotron radiation severely limits this technique too a few high end research activities.
Answer: We thank the reviewer for the detailed revisions and remarks. We included “synchrotron based” in the abstract.
